# Interventional Radiology Awareness Among Family Physicians and General Practitioners in the Qassim Region, Saudi Arabia

**DOI:** 10.7759/cureus.51751

**Published:** 2024-01-06

**Authors:** Abdulaziz Algharras, Husam Aloraini, Ahmad Aldosary, Ali Alsuhaibani, Abdullah N AlHwiriny, Omar Aloraini, Abdullah Alsuhibani, Sharifa Alduraibi, Asim S Aldhilan, Mohammed S Aldamegh

**Affiliations:** 1 Department of Radiology, College of Medicine, Qassim University, Buraydah, SAU; 2 College of Medicine, Qassim University, Buraydah, SAU; 3 Department of Family and Community Medicine, College of Medicine, Qassim University, Buraydah, SAU

**Keywords:** qassim region, family physician, radiology, awareness, interventional radiology

## Abstract

Interventional radiology (IR) is a rapidly growing specialty and is increasingly developing new procedures and minimally invasive interventions; thus, it might be challenging for non-specialists to keep up with all the new findings of IR. In this study, we tried to determine the level of awareness of family physicians and general practitioners about IR in the Qassim Region, Saudi Arabia. A self-completed online questionnaire was distributed among family physicians in the Qassim Region, with a total of 197 respondents. We found that the overall awareness level of family physicians and general practitioners about IR in the Qassim Region was low, with only 56 (28.4%) having an overall good knowledge level regarding IR, while 141 (74.6%) had poor knowledge. A total of 85 (43.1%) of the study participants reported that they would greatly benefit from more education about IR. The results demonstrate that there is a significant knowledge gap among family and general physicians about IR. Therefore, we suggest that there needs to be more education about IR and its developing procedures to promote collaboration among family medicine physicians, general physicians, and interventional radiologists.

## Introduction

Interventional radiology (IR) is an emerging and rapidly evolving medical specialty that is making significant progress quickly through image-guided diagnostic and therapeutic procedures that are marginally invasive [1]. IR as a specialty comprises minimally invasive methods using various radiological techniques, in which image-directed techniques are capable of approaching most body organs.

We investigate here the awareness and knowledge of family medicine doctors about IR after doing a thorough literature review on the subject. We found one study conducted in a large Canadian annual scientific assembly where the participants were 213 family physicians, and it showed that 31% and 53% of the contributors had poor and adequate knowledge, respectively, about IR in particular [2]. Furthermore, we found a few additional studies that assessed the awareness of IR among medical students, and one of these studies was done over 719 final-year medical students in the Riyadh region and concluded that there was a poor level of knowledge of IR in 83% of the participants [3]. Another study done at the University of Hail on about 200 clinical year's students and medical interns concluded that 45% of the participants had poor knowledge of IR [4]. In addition, a study among final-year medical students in England demonstrated that the knowledge of IR was poor when compared to other specialties for the bulk of students (55.5%), and there wasn’t any formal IR teaching during medical school (81.4%) [1]. On the other hand, a study done in Saudi Arabia at Jazan University showed that among the 270 medical students in both clinical and preclinical stages, 60.8% of clinical students had efficient knowledge about IR, while 39.2% of preclinical students had efficient knowledge about IR [5]. Traditionally, the low quantity of IR exposure and teaching in the undergraduate curriculum is a weakness that results in a lower chance of advising and advocating IR procedures to people in need. Exposure of medical students to radiological courses was shown to increase awareness and knowledge of radiology [1].

Family physicians play an essential role in discussing, educating, and deciding treatment plans and referring patients, as they are the front line in providing primary and comprehensive services to patients, which may often include different IR procedures [2]. Despite the growing number of facilities providing IR services in Saudi Arabia, there are insufficient studies in the medical literature on physicians' awareness of IR, specifically in the Middle Eastern area. Therefore, we are looking to study the family medicine physicians' and general practitioners’ knowledge and awareness of the IR specialty in the Qassim Region, Saudi Arabia.

The study aims to achieve specific objectives. Firstly, it seeks to assess the awareness of family and general physicians regarding various IR procedures. Secondly, it aims to evaluate the knowledge of these physicians concerning the training prerequisites and the professional activity of interventional radiologists. Lastly, it aims to assess potential educational approaches that could effectively enhance the understanding of family and general physicians concerning IR.

## Materials and methods

A descriptive cross-sectional study was carried out using a self-administered electronic survey, which was distributed among family physicians and general practitioners using convenience sampling of the target population. The data were collected from October 2022 to March 2023. The study's inclusion criteria encompassed practicing family physicians and general practitioners actively engaged in healthcare within the Qassim Region. This involved individuals actively practicing medicine within their respective roles. Conversely, the exclusion criteria were designed to exclude family physicians and general practitioners who were not actively involved in medical practice within the region, ensuring a focus on actively practicing professionals for the study's parameters.

By employing an online sample size calculator [6], we calculated a required sample size of 197 participants from the total population of Qassim family physicians and general practitioners, which stands at 400 according to data provided by the Academy of Family Physicians in the Qassim Region. This specific number is essential to attaining statistically significant findings within a 95% confidence interval.

The survey employed for data collection was developed based on a previous study done by Mok et al., which was done on family physicians in Canada in a large yearly assembly [2], and since both the population and the survey were close to ours, we opted to choose it. Subsequently, it was modified to align with our objectives and the specific population of interest. This revised survey underwent a thorough review process, involving both an IR specialist and a family medicine consultant.

The questionnaire encompassed various aspects, including participants' awareness of training requirements for IR, their knowledge of IR procedures and clinical duties, and the recognition of IR as a subspecialty by the Saudi Commission for Health Specialties (SCHS). Additionally, participants were asked about their cooperation with IR and their favored methods for future education in this regard.

## Results

Personal characteristics of family physicians

A total of 197 family medicine physicians were included in the study. Table 1 displays the age distribution of participants, ranging from 24 to 54 years, with a mean age of 32.4 ± 6.4 years. Of the total, 117 (59.4%) were male. In terms of specialty, 58 (29.5%) were family medicine specialists, 43 (21.8%) were family medicine consultants, 80 (40.6%) were family medicine residents, and 16 (8.1%) were general practitioners. Furthermore, 50 participants (25.4%) had 0-1 year of practice, 82 (41.6%) had 2-5 years of practice, and 48 (24.4%) had 6-10 years of practice.

**Table 1:** Demographic characteristics of family physicians, Qassim Region, Saudi Arabia GP: general practitioner, FM: family medicine

Personal data	No	%
Age in years		
20-29	84	42.6%
30-39	82	41.6%
40+	31	15.7%
Gender		
Male	117	59.4%
Female	80	40.6%
Specialty		
GP	16	8.1%
FM resident	80	40.6%
FM specialist	58	29.5%
FM consultant	43	21.8%
How many years have you been practicing (after your last training program)?		
0-1	50	25.4%
2-5	82	41.6%
6-10	48	24.4%
>10	17	8.6%

General knowledge of IR and specific IR procedures

Table 2 demonstrates the general knowledge of IR and specific IR procedures among the participants surveyed, where it shows that 34% ranked their knowledge of vascular and IR in comparison to other disciplines as good to excellent. Regarding specific procedures performed by IR, the findings were as follows: 60.9% reported familiarity with vascular angioplasty, 51.3% with varicose vein treatment, 50.3% with uterine fibroid embolization, 48.7% with radiofrequency ablation of tumors, and 42.6% with nephrostomy tube placement. However, 52% incorrectly identified IR's role in coronary angiography, 45% in vascular bypass procedures, and 38% in brain biopsy.

**Table 2:** General knowledge of IR and specific IR procedures IR: interventional radiology

	Awareness items	No	%
How would you rate your knowledge of vascular and IR when compared to other disciplines?	Poor	54	27.40%
	Adequate	76	38.60%
	Good	49	24.90%
	Excellent	18	9.10%
An interventional radiologist performs the following procedures: image-guided procedures			
Uterine fibroid embolization	Yes	99	50.30%
	No	26	13.20%
	Don’t know	72	36.50%
Coronary angiography	Yes	102	51.80%
	No	54	27.40%
	Don’t know	41	20.80%
Vascular angioplasty (diabetic ischemic foot)	Yes	120	60.90%
	No	33	16.80%
	Don’t know	44	22.30%
Radiofrequency ablation of tumors	Yes	96	48.70%
	No	33	16.80%
	Don’t know	68	34.50%
Peripheral vascular bypass procedure	Yes	89	45.20%
	No	53	26.90%
	Don’t know	55	27.90%
Brain biopsy	Yes	75	38.10%
	No	58	29.40%
	Don’t know	64	32.50%
Nephrostomy tube placement	Yes	84	42.60%
	No	48	24.40%
	Don’t know	65	33.00%
Varicose vein treatment	Yes	101	51.30%
	No	49	24.90%
	Don’t know	47	23.90%
Cystoscopic tumor resection	Yes	59	29.90%
	No	62	31.50%
	Don’t know	76	38.60%

Knowledge of IR training requirements, clinical duties, and collaboration with interventional radiologists

As Table 3 demonstrates, 32.5% of the family physicians knew that interventional radiologists received most of their training in radiology. A total of 55.8% knew that interventional radiologists had hospital admitting privileges, and 37.1% knew that they had outpatient clinics. Likewise, 58.4% of the participants knew that the Saudi Commission for Health Specialties (SCHS) recognizes vascular and IR as a distinct subfield of diagnostic radiology. A total of 29.4% referred a patient directly or indirectly to an interventional radiologist, and 58.9% reported that they would refer patients directly or indirectly to an interventional radiologist if they had more knowledge.

**Table 3:** Knowledge of IR training requirements, clinical duties, and collaboration with interventional radiologists SCFH: Saudi Commission for Health Specialties, IR: interventional radiology

Knowledge items		No	%
Interventional radiologists have received the majority of their training in	Radiology	64	32.50%
	Vascular surgery	23	11.70%
	Both	110	55.80%
Intervention radiologists have hospital admitting privileges	Yes	110	55.80%
	No	34	17.30%
	Don’t know	53	26.90%
Intervention radiologists have outpatient clinics	Yes	73	37.10%
	No	70	35.50%
	Don’t know	54	27.40%
Does the SCHS recognize vascular and IR as a distinct subspecialty of diagnostic radiology?	Yes	115	58.40%
	No	27	13.7%
	Don’t know	55	27.90%
Have you ever referred a patient directly or indirectly to an interventional radiologist?	Yes	58	29.40%
	No	139	70.60%
If you had more knowledge about IR, would you refer patients directly or indirectly to an interventional radiologist?	Yes	116	58.90%
	No	43	21.8%
	Don’t know	38	19.30%

Table 4 expresses that 85 (43.1%) of the study participants said that they would very much benefit from more education about interventional radiologists, while 21 (10.7%) thought they would not benefit at all. As for preferred methods for future education on IR, the most reported included interventional radiologists giving presentations at family medicine conferences (70.1%), followed by published articles in family physician journals (45.2%), self-directed learning websites (40.6%), and problem-based small group learning (35.5%).

**Table 4:** Interest in future education in IR and preferred methods of education IR: interventional radiology

Education items	No	%
How much would you benefit from more education about IR?		
Very much	85	43.10%
Somewhat	91	46.20%
Not at all	21	10.70%
The best method for future education on IR		
Interventional radiologists giving presentations at family medicine conferences	138	70.10%
Published articles in family physician journals	89	45.20%
Self-directed learning website	80	40.60%
Problem-based small-group learning	70	35.50%

Table 5 shows that a total of 51.6% of the family physicians aged 40 years or more had an overall good awareness level regarding IR compared to 20.2% of others aged 20-29 years with recorded statistical significance (p=0.004). Also, 35% of male physicians had a good awareness level versus 18.8% of females (p=0.013). Good awareness of IR was noticed among 41.2% of those with experience exceeding 10 years, compared to 18.3% of others with experience of two to five years (p=0.037). Additionally, 38.8% of those who gained very much benefit from more education about interventional radiologists had an overall good awareness in comparison to none of those who had no benefit at all (p=0.001).

**Table 5:** Factors associated with family physician's awareness of IR, Qassim Region, Saudi Arabia GP: general practitioner, FM: family medicine, IR: interventional radiology, * statistically significant

Factors	Poor		Good		p-value
	No	%	No	%	
Age in years					
20-29	67	79.80%	17	20.20%	0.004*
30-39	59	72.00%	23	28.00%	
40+	15	48.40%	16	51.60%	
Gender					
Male	76	65.00%	41	35.00%	0.013*
Female	65	81.30%	15	18.80%	
Specialty					
GP	11	68.80%	5	31.30%	
FM resident	62	77.50%	18	22.50%	
FM specialist	6	60.00%	4	40.00%	
FM register	33	68.80%	15	31.30%	
FM consultant	29	67.40%	14	32.60%	
How many years have you been practicing (after your last training program)?					
0-1	35	70.00%	15	30.00%	
2-5	67	81.70%	15	18.30%	0.037*
6-10	29	60.40%	19	39.60%	
>10	10	58.80%	7	41.20%	
How much would you benefit from more education about IR?					
Very much	52	61.20%	33	38.80%	0.001*
Somewhat	68	74.70%	23	25.30%	
Not at all	21	100.00%	0	0.00%	

## Discussion

Diagnostic radiology encompasses a group of subspecialties, such as IR, neuroradiology, pediatric radiology, and nuclear radiology [7,8]. In 2012, the American Board of Medical Specialties categorized IR as a separate specialty with an independent residency program [9]. The field of IR includes many practices such as central and peripheral arterial diseases, tumor and bleeding embolization, non-vascular procedures like drainage, biopsy and ablation, and treatment of venous vascular disease, among many other interventions to cover different organ systems [8].

This study aimed to assess family physicians' awareness of IR and related duties, and it showed that more than one-fourth (28%) of the physicians had an overall good awareness level of IR. Generally, about one-third (34%) of the physicians ranked their knowledge of vascular and IR when compared to other disciplines as good to excellent. A lower awareness level was reported by Mok et al. [2] who found that 31% of family doctors described their knowledge of IR as impoverished, 53% as acceptable, 14% rated as reasonable (14%), and only 2% rated as outstanding. Alnajjar et al. [10] revealed that 36.7% of medical interns at a Saudi university rated their knowledge of IR as poor, and only 15.7% reported that they knew nothing about IR. In the United Kingdom, work done by Muzumdar et al. [11] on undergraduates revealed a similar result. In relation to our research, these findings are below ours since only 27.4% of our population rated their knowledge as poor. Another paper done by Atiiga et al. [1] on final-year medical students showed that more than half of the students (55.5%) felt that their knowledge when compared to other specialties was poor, and this was also found in the study by O’Malley et al. [12]. The implication could be drawn from the papers by Atiiga et al. in 2017 and O’Malley et al. in 2012 [1,12], suggesting an improvement in knowledge of IR and teaching activities related to IR after those respective studies. This presumption arises given that Muzumdar et al.'s paper [11] was published in 2019. However, exploring and conducting further studies and research are necessary to uncover the actual causative factors behind these observed changes.

Regarding procedures performed by IR practitioners, approximately two-thirds (60.9%) affirmed the undertaking of vascular angioplasty, while around half (51.3%) acknowledged involvement in varicose vein treatment. Additionally, 50.3% confirmed engagement in uterine fibroid embolization, whereas less than half indicated participation in radiofrequency ablation of tumors (48.7%) and nephrostomy tube placement (43.6%). Conversely, approximately one-third (29.4%) denied involvement in a brain biopsy. Mok et al. [2] discovered that nearly all Canadian family physicians (98%) exhibited significantly greater knowledge regarding biopsies compared to less than 30% of our surveyed population. Furthermore, 71% of respondents in Mok PS et al.'s study [2] reported conducting uterine artery embolization, a higher proportion than the 50% observed in our sample. Both cohorts displayed similar accuracy rates of slightly less than 50% in understanding tumor ablation. Notably, over 60% of Saudi family physicians demonstrated better knowledge compared to 38% of the Canadian cohort in correctly recognizing vascular angioplasty as a procedure performed by interventional radiologists. This finding aligns closely with Atiiga PA et al.'s study [1], wherein 75% of final-year medical students identified vascular angioplasty as a procedure within the purview of IR.

In terms of IR training prerequisites, clinical responsibilities, and collaboration with interventional radiologists, about one-third (32.5%) of participants were aware that interventional radiologists received most of their training in radiology. Conversely, in Mok et al.'s Canadian study [2], approximately 76% of the subjects knew about this training background. Additionally, we found that more than half of our participants (55.8%) knew that interventional radiologists possess hospital admitting privileges, which is much higher than 27% in O’Malley et al. [12]. Only one-third of our participants (37.1%) were aware of their outpatient clinics. Similarly, more than half of the participants (58.4%) are aware that the SCHS recognizes IR and vascular imaging as distinct subfields of diagnostic radiology.

Regarding educational aspects, this study revealed that less than half of the participants (43.1%) believed they would greatly benefit from more education about IR, with a smaller percentage (10.7%) thinking they would not benefit at all. When it comes to preferred methods for future education on IR, the most commonly reported options include interventional radiologists speaking at conferences for family medicine (70.1%), followed by published papers in publications for family physicians (45.2%), independent learning websites (40.6%), and problem-focused group learning (35.5%). These findings align with Mok et al.'s [2] results, who reported that most family physicians believed that future training in IR would be extremely or kind of beneficial. Roughly 43% of them chose that interventional radiologists speaking at family medicine conferences represent their selected means of continuing education. Similarly, Alnajjar et al. [10] found that 61.4% of medical students consider a necessary two-week IR rotation as a component of the medical school curriculum for surgery to be helpful. These findings are consistent with a Canadian study done by O’Malley et al. [12], where approximately 71% of students supported the idea of a necessary two-week IR rotation.

Several limitations characterize this study. Primarily, its scope within the Qassim Region implies that the findings may not entirely represent Saudi family physicians or general practitioners on a national scale. Moreover, participants self-assessed their knowledge of IR in the initial segment of the "general knowledge of IR and specific procedures" questionnaire. The study employed convenience sampling as its sampling technique. Lastly, it lacked the capacity to provide an educational supplementary video or article. This absence hindered the ability to gauge whether an intervention would have elevated the knowledge rate. It also precluded the exploration of potential avenues for further research based on the outcomes of such an intervention.

We suggest a focus on implementing an intervention and assessing the knowledge levels and perspectives of family physicians both before and after its implementation. This approach aims to ascertain whether it can induce substantial and sustainable changes in knowledge, ultimately facilitating the delivery of appropriate care to patients.

## Conclusions

The study demonstrates that there is a gap in the knowledge of family physicians and general practitioners in the Qassim Region about IR. Many of the family physicians believe that they would have benefited from more education about IR. The knowledge gap might be tackled by providing good, clinical-based curricula in family physician rotation training. However, more research would be needed to suggest practical solutions to close this knowledge gap and help them discuss with their patients different treatment modalities that might be less invasive than surgery and more curative than conservative management. We suggest doing more extensive studies on the most appropriate teaching modality to push the appropriate, effective care to reach the patient.

## Appendices

Survey of family physicians on their awareness of IR

❑ I confirm that I have read and understood the procedures in this research and I have agreed to them, and I am ready to participate in this survey

Demographics

1) What stage are you at in your medical career (consultant, register…)?

……………………

2) Gender

❑Male

❑Female

3) What is your age?

…………………….

4) How many years have you been practicing (after your last training program)?

………………….

General Knowledge of IR and Specific IR Interventions

5) How would you rate your knowledge of vascular and interventional radiology when compared to other disciplines?

❑Poor             ❑Adequate       ❑Good          ❑Excellent

6) An interventional radiologist performs

i) Uterine fibroid embolization

❑Yes               ❑No   ❑Don‘t know

ii) Coronary angiography

❑Yes               ❑No   ❑Don‘t know

iii) Vascular angioplasty (diabetic ischemic foot)

❑Yes               ❑No   ❑Don‘t know

iv) Radiofrequency ablation of tumors

❑Yes               ❑No   ❑Don‘t know

v) Peripheral vascular bypass procedure

❑Yes               ❑No                ❑Don‘t know

vi) Brain biopsy

❑Yes               ❑No                ❑Don‘t know

vii) Nephrostomy tube placement

❑Yes               ❑No                ❑Don‘t know

viii) Varicose vein treatment

❑Yes               ❑No                ❑Don‘t know

ix) Cystoscopic tumor resection

❑Yes               ❑No                ❑Don‘t know

Awareness of IR Training and Activities

8) Interventional radiologists have received the majority of their training in

❑Radiology     ❑Vascular Surgery                 ❑Both

9) An interventional radiologist has

i) Hospital admitting privileges

❑Yes               ❑No   ❑Don‘t know

ii) Outpatient clinics

❑Yes               ❑No   ❑Don‘t know

10) Does the Saudi Commission for Health Specialties (SCHS) recognize vascular and interventional radiology as a distinct subspecialty of diagnostic radiology?

❑Yes               ❑No   ❑Don‘t know

11) Have you ever referred a patient directly or indirectly to an interventional radiologist?

❑Yes               ❑No

12) If you had more knowledge about interventional radiology, would you refer patients directly or indirectly to an interventional radiologist?

 ❑Yes            ❑No     ❑Don‘t know

Preferred Educational Method for Further IR Knowledge

13) How much would you benefit from more education about interventional radiologists?

❑Not at all      ❑Somewhat    ❑Very much

14) What would be the best method for future education on interventional radiology (you can select more than one response)?

❑ Interventional radiologists giving presentations at family medicine conferences

❑ Published articles in family physician journals

❑ Self-directed learning website

❑ Problem-based small-group learning

15) Additional comments (optional)

…………………………………………………………………………………………………..

…………………………………………………………………………………………………..

…………………………………………………………………………………………………..
