# Correction: Interventional Radiology Awareness Among Family Physicians and General Practitioners in the Qassim Region, Saudi Arabia

**DOI:** 10.7759/cureus.c165

**Published:** 2024-03-12

**Authors:** Abdulaziz Algharras, Husam Aloraini, Ahmad Aldosary, Ali Alsuhaibani, Abdullah N AlHwiriny, Omar Aloraini, Abdullah Alsuhibani, Sharifa Alduraibi, Asim S Aldhilan, Mohammed S Aldamegh

**Affiliations:** 1 Department of Radiology, College of Medicine, Qassim University, Buraydah, SAU; 2 College of Medicine, Qassim University, Buraydah, SAU; 3 Department of Family and Community Medicine, College of Medicine, Qassim University, Buraydah, SAU

This article has been corrected at the request of the authors to change the affiliations as follows:

Ahmad Aldosary Original: Department of Family and Community Medicine, Qassim University, Unaizah College of Medicine and Medical Sciences, Unaizah, SAU Corrected: Department of Family and Community Medicine, College of Medicine, Qassim University, Buraydah, SAU.

Abdulaziz Algharras Original: Department of Radiology, Qassim University, Unaizah College of Medicine and Medical Sciences, Unaizah, SAU Corrected: Department of Radiology, College of Medicine, Qassim University, Buraydah, SAU.

Sharifa Alduraibi Original: Department of Radiology, Qassim University, Buraydah, SAU Corrected: Department of Radiology, College of Medicine, Qassim University, Buraydah, SAU.

Asim S. Aldhilan Original: Department of Radiology, Qassim University, Buraydah, SAU Corrected: Department of Radiology, College of Medicine, Qassim University, Buraydah, SAU.

Mohammed S. Aldamegh Original: Department of Radiology, Qassim University, Unaizah College of Medicine and Medical Sciences, Unaizah, SAU Corrected: Department of Radiology, College of Medicine, Qassim University, Buraydah, SAU.

The authors deeply regret that these errors were not identified and addressed prior to publication.

 

